# The Best Method for Removing the Upper Maxillary Bone

**Published:** 1873-05

**Authors:** Julian J. Chisholm

**Affiliations:** Professor of Operative Surgery in the University of Maryland.


### ARTICLE VII.

### *The Best Method for Removing the Upper Maxillary Bone.*

BY JULIAN J. CHISHOLM, M. D.,

Professor of Operative Surgery in the University of Maryland.

As a surgeon, but more especially as a teacher of operative surgery, I have constantly occasion to consult authorities on the various methods adopted by surgeons for the performance of operations. In reviewing the *modus operandi* for extirpating the upper jaw, I have noticed that in the many works on practical surgery, even the most recent, but one mode of procedure is described. All of these books mention the curved incision through the cheek, extending from the external angular process of the frontal bone to the angle of the mouth; to be modified, if found necessary, by the addition of a horizontal incision running immediately beneath and parallel with the lower lid, and extending from below the inner canthus of the eye to the vertical cheek incision. If required, this horizontal incision may be made mid-way between the mouth and the orbit, extending from the wing of the nose directly outward to the curved incision already referred to. Mr. Ferguson had suggested an incision passing through the median line of the upper lip, then curving around the wing of the nostril and extending upwards in the line where the nose and cheek meet each other to within one-half or three-quarters of an inch of the inner canthus of the eye. When only a portion of the upper jaw is to be removed, this latter incision around the nose appears to be sufficiently extensive for exposing the surface. When a larger surface is to be laid bare, Mr. Fergusson adds this nasal incision to the curved incision extending from the angle of the mouth upwards. This curved incision through the side of the face is open to the very serious objection of leaving an ugly scar, to which is added paralysis of all of the muscles in front of the wound through division of the branches of the facial nerve. When the horizontal incisions are used, the face is much more extensively scarred and thereby permanently disfigured.

Having for many years taught and practised Dieffenbach's operation for the removal of the upper jaw as the best method, both as the ready exposure of the entire bone and leaving the least deformity as a permanent result, I have been much surprised to find so little mention of this excellent method. Chelius, in his System of Surgery, refers to Dieffenbach's method in one line only. In the more recent works of Holmes, Erichson, Fergusson, Gross, Gant, and others, the operation appears to have been completely lost sight of.

Dieffenbach's operation consists in making the incision in the median line of the face. Commencing at the root of the nose, an incision slits the nose and the upper lip in the median line. A short incision joining the first at right angles, extends from the root of the nose to the inner angle of the eye. The lower line being drawn downwards, the knife is carried along the entire length of the conjunctival cul-de-sac, separating this lid from its orbital connection, and utilizing the entire length of the lower lid in the horizontal flap. When the flap, as defined by the vertical and horizontal incision, is dissected up, it will lay bare the entire front, and, if necessary, side of the face, without having divided any large vessel, or any important nerve branch. With such an exposure the superior maxillary bone can be isolated with great ease as every surface of contact with neighboring bones can be clearly brought into view. With no additional incision I found no difficulty in removing from the living subject the superior maxilla, molar, and palate bone, which enabled me to extirpate a large fibroid with extensive adhesions to the roof of the pharynx. After the removal of the maxilla, when the flap is brought back to its normal situation and carefully adjusted by several points of suture, union speedily ensues. This operation leaves so little deformity that in the majority of cases the line of incision will escape detection unless the scar be sought. In many faces the thin skin is so stretched over the bridge of the nose as a normal condition, that it reflects a light as a whitish line somewhat similar to the scar made in this operation. The inferior lid retains all of its movements. The sides of the face present their normal appearances unscarred, with nerves and vessels intact. The eye-muscles can be so nicely manipulated through this bold flap, that all the movements are retained. As the suspensory ligaments of the eye-ball remain adherent to the roof and side of the orbit, there is no drooping of the eye-ball. Immediately after the suture are placed I have seen the patient move the eyes in parallel axis, showing perfect control.

Experience both on the living and dead subject has proved to me the advantages which Dieffenbach's operation possesses over all others designed for the removal of the entire upper jaw. I would recommend to those who may have this operation to perform, to give the method by median vertical incision a trial, feeling assured that those who have once used this better method will not go back to the incision through the cheek into the angle of the mouth, as described in most treatises on surgery.—*Med. Record.*

